# A study of community structure and beta diversity of epiphyllous liverwort assemblages in Sabah, Malaysian Borneo

**DOI:** 10.3897/phytokeys.153.53637

**Published:** 2020-07-16

**Authors:** Tamás Pócs, Gaik Ee Lee, János Podani, Elizabeth Pesiu, Judit Havasi, Hung Yung Tang, Andi Maryani A. Mustapeng, Monica Suleiman

**Affiliations:** 1 Eszterházy University, Institute of Biology, Botany Department, Eger, Pf. 43, H-3301, Hungary; 2 Faculty of Science and Marine Environment, 21030, Kuala Nerus, Universiti Malaysia Terengganu, Terengganu, Malaysia; 3 Institute of Tropical Biodiversity and Sustainable Development, 21030, Kuala Nerus, Universiti Malaysia Terengganu, Terengganu, Malaysia; 4 Department of Plant Systematics, Ecology and Theoretical Biology, Eötvös University, H-1117, Budapest, Hungary; 5 Balassi Institute, Bajcsy-Zsilinszky street 57. III. 1065, Budapest, Hungary; 6 Department of Geology, University of Malaya, 50603, Kuala Lumpur, Malaysia; 7 Forest Research Centre, Sabah Forestry Department, PO Box 1407, 90715, Sandakan, Sabah, Malaysia; 8 Institute for Tropical Biology and Conservation, Universiti Malaysia Sabah, 88400, Kota Kinabalu, Sabah, Malaysia

**Keywords:** Lejeuneaceae, liverworts, Malesia, Marchantiophyta, statistical analyses

## Abstract

We evaluated the species richness and beta diversity of epiphyllous assemblages from three selected localities in Sabah, i.e. Mt. Silam in Sapagaya Forest Reserve, and Ulu Senagang and Mt. Alab in Crocker Range Park. A total of 98 species were found and a phytosociological survey was carried out based on the three study areas. A detailed statistical analysis including standard correlation and regression analyses, ordination of species and leaves using centered principal component analysis, and the SDR simplex method to evaluate the beta diversity, was conducted. Beta diversity is very high in the epiphyllous liverwort assemblages in Sabah, with species replacement as the major component of pattern formation and less pronounced richness difference. The community analysis of the epiphyllous communities in Sabah makes possible their detailed description and comparison with similar communities of other continents.

## Introduction

Beta-diversity can be defined as the change or turnover in species composition among particular sites (Anderson et al. 2011). This pattern provides a platform into understanding processes that form and maintain biodiversity (e.g., Tuomisto et al. 2003; Chase 2010; Anderson et al. 2011; Kraft et al. 2011). According to Whittaker (1972), the level of beta-diversity in plant communities is associated with two mechanisms known as habitat heterogeneity and dispersal limitation. This has brought the attention of ecologists to further assess the patterns of beta-diversity and to investigate the mechanisms behind observed patterns through specifically designed data collection (Smith 1982; Bolnick et al. 2002; Harrison et al. 2006; Philippot and Hallin 2011; Myers et al. 2013). Hence, epiphyllous liverworts communities seem to give advantages and provide an excellent system for the study of beta diversity (Kraichak 2014) in numerous ways. First, they can be easily sampled and obtained in a large number within a relatively small area and extended across multiple habitat types and scales (Kraichak 2014). They usually occur and thrive well in moist and warm forests of tropical and subtropical regions (Chen and Wu 1964) and can be preserved intact for later examination (Richards 1932). Besides, due to their simple morphological structure and poikilohydric status, they rely greatly on air moisture as the condition of survival, allowing reliable quantification of particular resource levels and fluctuations (Monge-Najera 1989; Pócs 1996; Gradstein 1997; Pócs and Tóthmérész 1997; Zotz et al. 1997).

Liverworts commonly occur as epiphytes and epiphylls in tropical rainforests (Gradstein 1997; Gehrig-Downie et al. 2013). The epiphylls or epiphyllous liverworts (i.e. species found growing on the living leaves of vascular plants) constitute a special life form, occurring in permanently moist and warm evergreen forests in tropical and subtropical regions. They are considered as the most important component in epiphyllous assemblages, in which an average of 4–8, but sometimes much more, up to 25 species, can grow on a single leaf (Lücking 1995; Gehrig-Downie et al. 2013). In addition, they often exhibit high rates of endemism, especially in montane forests above 1,500 m elevation (Pócs 1996). Epiphyllous liverworts have been described since the 18^th^ century; the first report of an epiphyllous liverwort, i.e., *Jungermannia flava* Sw. (= *Lejeunea flava* (Sw.) Nees), was given by Swartz in 1788. Since then, epiphyllous liverworts have attracted and captured the interest of numerous botanists and ecologists because of their unique habitat, their life strategies, and adaptations necessary for surviving in such microhabitat (Goebel 1890; Ruinen 1961; Winkler 1967, 1970; Pócs 1996; Sonnleitner et al. 2009). About one thousand species of epiphyllous bryophytes have been described. Apparently, they have certain morphological characters which allow them to colonize and survive in this ephemeral environment. Epiphylls have long been recognised as the phyllosphere of vascular plant communities (Ruinen 1961). Several studies have been conducted on morphological and life-history characters related to the survival of epiphylls and the correlation of microclimatic variables with the distribution of epiphyllous communities (Gradstein 1997; Gignac 2001; Wanek and Pörtl 2005; Frego 2007; Sonnleitner et al. 2009; Hylander et al. 2013; Malombe et al. 2016).

Sabah, located at the East of Malaysia, consists of several unique landscapes and regions of higher altitudes that offer promising biological sites for the study of epiphyllous liverworts. Much of this region has been declared either as state parks under the management of Sabah Parks or conservation areas under the management of Yayasan Sabah Group. For example, the Crocker Range, the longest range in Sabah extending from Kudat (northern tip of Borneo) to Sipitang (southern part of Sabah) (Suleiman et al. 2017), has the highest mountain peak in Southeast Asia (Mount Kinabalu, 4059 m a.s.l), together with other 16 peaks that exceed 1,000 m above sea level (Usui et al. 2006). Meanwhile, huge areas of unique landscapes (basin, valley, coast, canyon and river) that have been protected host a remarkable biological diversity with a staggering number of plant species.

A fair number of bryophyte studies have been published and reported from Sabah (e.g., Mizutani 1974; Inoue 1989; Yamada 1989; Piippo 1989; Frahm et al. 1990; Akiyama et al. 2001; Suleiman et al. 2006; Andi et al. 2015; Zhu et al. 2017). However, no specific study focused on epiphyllous liverwort communities has been conducted in tropical rainforests of Sabah and within Malaysia. Therefore, the present study is aimed to evaluate the species richness and beta diversity of epiphyllous assemblages from three selected localities in Sabah by performing a phytosociological survey and detailed statistical analysis.

## Materials and methods

### Study area

**1) Ulu Senagang**

**Figure 1.**
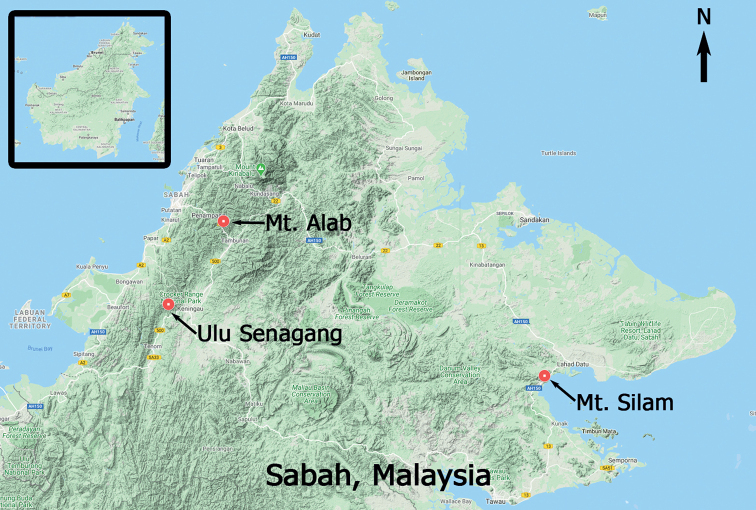
The three selected localities in the present study.

Ulu Senagang is located in the western part of Sabah (Fig. 1), near the boundary of Tenom and Keningau districts. It is part of the Crocker Range Park (CRP) and located in the south eastern zone of the park. The CRP was shaped by the Crocker Range Formation where the lower part is of Paleocene to Middle Eocene age (Hutchison 2005). The most dominant parental soil types found in the Crocker Range are sandstone and mudstone (Dinor et al. 2007). The temperature on the lowlands of CRP is within 22–40 °C throughout the year. CRP has one of the highest precipitation areas in Sabah. However, the eastern part of the park, including Ulu Senagang, has a relatively low rainfall with less than 2,000 mm/year (Usui et al. 2006). The forest vegetation zone of Ulu Senagang is lowland rainforest and it is classified as hill dipterocarp forest. According to Majit et al. (2011), the forest type of this area is considered as a young secondary forest due to past disturbance from human activities and forest fires.

**2) Mount Silam**

Mount Silam is a small coastal mountain located at the south-eastern part of Sabah in Lahad Datu district (Fig. 1). Most of the mountain is made up of ultrabasic rock. Standing at only 884 m a.s.l., this mountain experiences frequent cloud cap formation which usually develops from the early afternoon until the end of the day. The forest above 770 m is stunted, showing a classic ‘Massenerhebung effect’, which is the compression of forest zones on a small mountain (Proctor et al. 1988). The altitudinal gradient of Mount Silam can be divided into four layers which are the lowland ultramafic forest (200–300 m), upland ultramafic forest (330–540 m), lower montane ultramafic forest (540–770 m) and the upper montane ultramafic forest (>770 m) (Sabah Forestry Department 2017). The lowland climate of Mount Silam is humid tropical with an average precipitation of 2,132 mm/year. The annual mean temperature is 27 °C and the mean monthly relative humidity is about 85%. However, the summit region receives higher rainfall of up to 2,700 mm/year and relative humidity of 90–91% (Bruijnzeel et al. 1993). The mean temperature of the summit region is 18.8–27.7 °C (Proctor et al. 1988).

**3) Mount Alab**

Mount Alab is located in the northern zone of the Crocker Range Park in Tambunan district. This area shares the same geological formation and soil types with Ulu Senagang. This mountain is the second highest peak of CRP with 1964 m a.s.l. The forest vegetation zone of this area is upper montane rainforest, called also “cloud- or “mossy-forest”. It is classified as a primary forest and dominated by montane plants from the Fagaceae, Myrtaceae and Ericaceae. Mount Alab receives the highest rainfall in CRP with more than 4000 mm/year. The mean air temperature and relative humidity of this mountain are about 15 °C and 99%, respectively (Majuakim and Anthony 2016). The peak of Mount Alab is persistently covered with clouds from mid-day, resulting in high abundance of bryophytes.

### Sampling and data analysis

During our present study in Malaysia, by the selection and guidance of the second author, we studied 23 rainforest habitats in Sabah. Of these we could take representative samples of the epiphyllous communities in 12 habitats at different altitudes. The routine followed the sampling protocol of Pócs (1978). For the present study, we selected three sites: Crocker Range Park, W of Keningau district at Ulu Senagang Substation (a lowland rainforest at 525–570 m elevation); Crocker Range Park, NNW of Tambunan district at Gunung Alab Substation (mossy elfin forest or cloud forest at 1900–1940 m elevation); and Mt. Silam, Sapagaya Forest Reserve of Lahad Datu district (lower montane rainforest at 600–740 m elevation). From the shrub layer of each site, 50 average sized leaves well-covered by epiphylls were collected randomly and prepared for further study. From a coenological point of view, each leaf was considered to be a different stand of the epiphyllous assemblage. The species composition on each leaf was identified, yielding a total of 98 species. That is, the present study is based on a 98 × 150 presence-absence data matrix, as given separately for the three study areas in Tables 2–4. In addition, the area of each leaf was also measured.

The epiphyllous liverwort assemblage data served as a basis for a detailed statistical survey. The relationship between the number of species and leaf area was graphically illustrated by a scatterplot, and standard correlation and regression analyses were conducted to evaluate its linear component. Centered (i.e. covariance-based) principal component analysis (Podani 2001) was used to generate a simultaneous ordination of species and leaves, the biplot. In addition, beta diversity and related structural phenomena were evaluated by the SDR simplex method developed by Podani and Schmera (2011).

## Results and discussion

### The localities of epiphyllous collections and phytosociological survey

Table 1 shows the enumeration of rainforest habitats visited during the period of 30 July to 17 August, 2018 in which epiphyllous liverworts were collected. In Tables 2–4, each column represents the epiphyll flora of one leaf. The leaf area in cm^2^ and the number of species of each leaf are indicated. The X and + symbols mean presence only, while the black dots in Table 3 indicate the dominant species on each leaf. The species are arranged according to their frequency in the analysed communities. Table 5 shows the comparison of the three assemblages, their similarities and differences, in which species with at least 10% occurrence in Tables 2–4 are included only. Those with frequency less than 5 out of 50 are omitted.

**Table 1.** The three investigated habitats in the present study.

Locality		Forest type	GPS coordinates	Elevation (m)
**Table 2.**	**1822.** Ulu Senagang Substation, Crocker Range Park, Keningau district	Lowland rainforest below waterfalls, with 50 m high canopy of Dipterocarpaceae	05°21.776'N, 116°01.713'E	525–570
**Table 3.**	**1811.** Mt. Silam, Sapagaya Forest Reserve, 22 km WSW of Lahad Datu district, from the telecommunication towers to the summit ridge of Mt. Silam	Lower montane rainforest with 15–20 m high canopy with *Shorea tenuiramulosa* and *Borneodendron enigmaticum*.	04°57'12"N, 118°9'39"E	600–740
**Table 4.**	**1823.** Mt. Alab Substation, Crocker Range Park, Tambunan district	Mossy cloud (elfin) forest, about 6 m high canopy of *Phyllocladus hypophyllus*, *Rhododendron*, *Dacrydium* and *Nepenthes* spp.	05°49.320'N, 116°20.499'E.	1900–1940

### Species/leaf area relationships and beta diversity analyses

**Figure 2.**
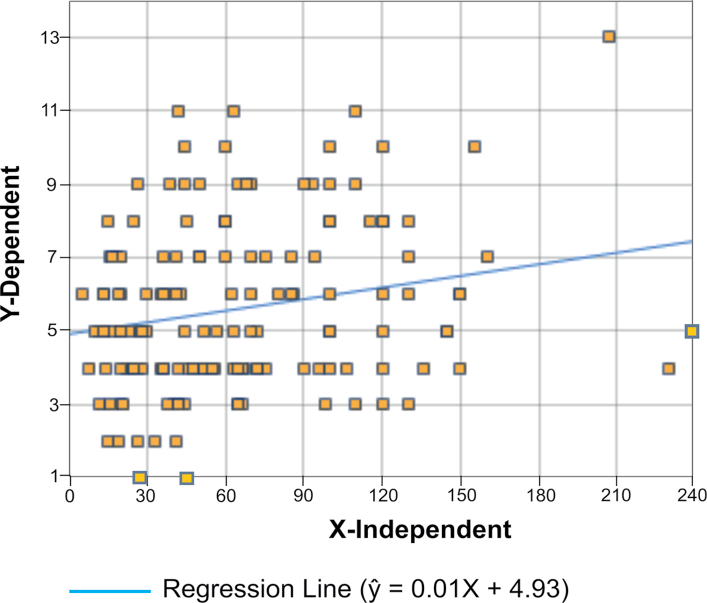
Leaf area (in cm^2^, x axis) – number of epiphyll liverwort species (y axis) relationship based on 150 leaves collected in three rainforest sites in Sabah.

The number of species vs leaf area relationships are shown by the scatter plot in Fig. 2. Although the variance of the number of species per leaf is fairly high, there is a definite increase of species number over area. Since the number of points is large, and therefore the degrees of freedom is also large (n = 148), the resulting Pearson correlation, *r* = 0.22 with a probability point of p = 0.007, is a highly significant result. The regression equation is *N* = 0.01*A* + 4.93 in which *N* is the estimate of species number at leaf area *A* expressed in cm^2^.

**Table 2.** The epiphyllous communities in Ulu Senagang, a lowland tropical rainforest at 525–570 m elevation.

**Leaf surface area in cm^2^**	**130**	**44**	**120**	**150**	**38**	**145**	**115**	**46**	**17**	**70**	**120**	**30**	**42**	**96**	**45**	**240**	**130**	**12**	**230**	**14**	**44**	**13**	**85**	**41**	**70**	
**Cover of epiphylls in % of leaf surface**	**5**	**25**	**70**	**2**	**20**	**10**	**40**	**4**	**15**	**30**	**18**	**7**	**12**	**18**	**40**	**9**	**5**	**55**	**8**	**40**	**50**	**65**	**8**	**18**	**20**	
**Species number of each leaf**	**8**	**5**	**10**	**4**	**6**	**5**	**8**	**4**	**5**	**9**	**8**	**6**	**4**	**4**	**8**	**5**	**6**	**3**	**4**	**4**	**3**	**5**	**7**	**2**	**6**	
*Leptolejeunea epiphylla* (Mitt.) Steph.	.	.	X	X	X	X	X	.	X	.	X	.	.	X	X	.	.	.	X	X	X	.	X	.	X	
*Leptolejeunea maculata* (Mitt.) Schiffn.	X	X	.	X	X	.	X	X	X	X	X	X	X	X	.	X	.	.	X	X	.	.	X	X	.	
*Cololejeunea planissima* (Mitt.) Abeyw.	X	.	X	X	X	X	.	.	.	X	X	.	.	.	X	X	X	X	X	X	.	.	.	.	X	
*Cololejeunea gottschei* (Steph.) Mizut.	X	.	.	.	X	.	.	.	X	X	.	X	X	.	.	X	X	X	.	X	.	.	X	.	X	
*Lejeunea* sp.	.	.	.	.	.	.	.	.	.	.	.	.	.	.	.	X	.	X	.	.	X	.	.	.	.	
*Drepanolejeunea tenera* K.I.Goebel	.	.	X	.	.	.	X	X	.	.	X	X	.	X	.	X	.	.	.	.	.	.	X	.	X	
*Cheilolejeunea trapezia* (Nees) R.M.Schust. & Kachroo	.	.	X	.	.	.	X	.	.	X	.	.	X	.	.	.	.	.	.	.	.	X	.	.	.	
*Cololejeunea lanciloba* Steph.	.	X	.	.	.	.	.	.	X	.	.	.	.	.	.	.	X	.	X	.	.	.	X	.	.	
*Cololejeunea longifolia* (Mitt.) Mizut.	.	.	.	.	.	.	.	X	.	X	X	.	.	X	.	.	.	.	.	.	.	.	.	.	.	
*Leptolejeunea vitrea* (Nees) Schiffn.	X	.	X	.	.	X	X	X	.	.	X	.	.	.	.	.	.	.	.	.	.	.	.	.	.	
*Microlejeunea punctiformis* Taylor (Steph.)	.	.	.	.	.	.	.	.	.	.	.	.	.	.	.	.	.	.	.	.	.	X	.	.	.	
*Cololejeunea hildebrandii* (Austin) Steph.	.	.	X	.	.	.	.	.	.	.	.	.	.	.	X	.	.	.	.	.	.	.	.	.	.	
*Cololejeunea peponiformis* Mizut.	.	.	.	.	.	.	.	.	.	X	.	.	.	.	X	.	.	.	.	.	.	.	.	.	.	
*Drepanolejeunea pentadactyla* (Mont.) Steph.	.	.	.	.	.	X	.	.	X	X	.	X	X	.	.	.	.	.	.	.	.	.	.	.	.	
*Lejeunea* sp.2	X	X	.	.	X	.	X	.	.	.	.	X	.	.	.	.	.	.	.	.	.	.	.	.	.	
*Caudalejeunea reniloba* (Gottsche) Steph.	.	.	.	.	.	.	.	.	.	.	.	X	.	.	.	.	X	.	.	.	.	.	X	.	.	
*Cololejeunea tenella* Benedix	.	X	.	.	X	.	X	.	.	X	.	.	.	.	.	.	.	.	.	.	.	.	.	.	.	
*Colura acroloba* (Steph.) Ast	.	.	X	X	.	.	X	.	.	X	.	.	.	.	.	.	.	.	.	.	.	.	.	.	.	
*Frullania* sp.	.	.	.	.	.	.	.	.	.	.	.	.	.	.	X	.	X	.	.	.	.	.	.	.	.	
*Lopholejeunea subfusca* (Nees) Schiffn.	.	.	.	.	.	.	.	.	.	.	X	.	.	.	.	.	.	.	.	.	.	X	.	.	.	
*Cololejeunea acuminata* Mizut.	X	.	.	.	.	.	.	.	.	.	.	.	.	.	.	.	.	.	.	.	.	.	.	.	.	
*Cololejeunea raduliloba* Steph.	.	.	.	.	.	.	.	.	.	.	.	.	.	.	.	.	.	.	.	.	X	.	.	X	X	
*Drepanolejeunea vesiculosa* (Mitt.) Steph.	.	.	.	.	.	.	.	.	.	.	.	.	.	.	.	.	.	.	.	.	.	X	.	.	.	
*Leptolejeunea elliptica* (Lehm. & Lindenb.) Schiffn.	.	X	.	.	.	X	.	.	.	.	.	.	.	.	.	.	.	.	.	.	.	.	X	.	.	
*Cheilolejeunea* (*Cyrtolejeunea*?)	X	.	X	.	.	.	.	.	.	.	.	.	.	.	.	.	.	.	.	.	.	.	.	.	.	
*Colura conica* (Sande Lac.) K.I.Goebel	X	.	X	.	.	.	.	.	.	.	.	.	.	.	.	.	.	.	.	.	.	.	.	.	.	
*Colura corynophora* (Nees et al.) Trevis	.	.	.	.	.	.	.	.	.	.	X	.	.	.	X	.	.	.	.	.	.	.	.	.	.	
*Leptolejeuna ligulata* Herzog	.	.	.	.	.	.	.	.	.	.	.	.	.	.	X	.	.	.	.	.	.	.	.	.	.	
*Leptolejeunea tripuncta* (Mitt.) Steph.	.	.	.	.	.	.	.	.	.	.	.	.	.	.	.	.	.	.	.	.	.	.	.	.	X	
*Cheilolejeunea intertexta* (Lindenb.) Steph.	.	.	.	.	.	.	.	.	.	.	.	.	.	.	.	.	.	.	.	.	.	.	.	.	.	
*Cheilolejeunea vittata* (G.Hoffm.) R.M.Schust & Kachroo	.	.	.	.	.	.	.	.	.	.	.	.	.	.	.	.	.	.	.	.	.	.	.	.	.	
Cololejeunea aff. schmidtii Steph.	.	.	.	.	.	.	.	.	.	.	.	.	.	.	.	.	.	.	.	.	.	.	.	.	.	
*Cololejeunea stylosa* (Steph.) Mizut.	.	.	.	.	.	.	.	.	.	.	.	.	.	.	.	.	X	.	.	.	.	.	.	.	.	
*Colura superba* (Mont.) Steph.	.	.	.	.	.	.	.	.	.	.	.	.	.	.	.	.	.	.	.	.	.	X	.	.	.	
*Colura ornata* K.I.Goebel	.	.	.	.	.	.	.	.	.	.	.	.	.	.	X	.	.	.	.	.	.	.	.	.	.	
*Lejeunea flava* (Sw.) Nees	.	.	X	.	.	.	.	.	.	.	.	.	.	.	.	.	.	.	.	.	.	.	.	.	.	
*Lopholejeunea nigricans* (Lindenb.) Schiffn.	.	.	.	.	.	.	.	.	.	.	.	.	.	.	.	.	.	.	.	.	.	.	.	.	.	
*Microlejeunea filicuspis* (Steph.) Heinrichs et al.	.	.	.	.	.	.	.	.	.	.	.	.	.	.	.	.	.	.	.	.	.	.	.	.	.	
**Leaf surface area in cm^2^**	**23**	**70**	**120**	**50**	**66**	**63**	**36**	**90**	**27**	**42**	**98**	**56**	**240**	**66**	**44**	**39**	**44**	**100**	**28**	**38**	**17**	**20**	**28**	**100**	**75**	**B**
**Cover of epiphylls in % of leaf surface**	**12**	**6**	**5**	**6**	**7**	**8**	**30**	**8**	**28**	**24**	**60**	**5**	**3**	**30**	**14**	**10**	**5**	**30**	**4**	**16**	**12**	**30**	**7**	**9**	**45**	**50**
**Species number of each leaf**	**5**	**5**	**6**	**4**	**4**	**4**	**6**	**8**	**1**	**3**	**3**	**4**	**5**	**3**	**9**	**9**	**10**	**5**	**4**	**3**	**7**	**3**	**5**	**5**	**4**	
*Leptolejeunea epiphylla* (Mitt.) Steph.	X	.	X	.	X	X	.	X	.	X	X	X	X	X	X	X	.	X	.	X	X	X	X	X	X	32
*Leptolejeunea maculata* (Mitt.) Schiffn.	X	X	X	.	X	X	.	X	.	X	.	.	X	.	X	X	X	.	.	.	X	.	X	X	X	32
*Cololejeunea planissima* (Mitt.) Abeyw.	.	X	X	.	.	.	.	.	.	.	.	.	.	.	.	X	.	X	X	X	X	.	.	X	.	22
*Cololejeunea gottschei* (Steph.) Mizut.	X	X	X	.	.	.	.	.	.	.	.	.	.	.	X	X	X	.	.	.	X	.	.	.	.	19
*Lejeunea* sp.	X	.	.	.	X	X	X	X	.	.	.	X	X	.	.	X	X	.	X	X	X	X	X	.	X	18
*Drepanolejeunea tenera* K.I.Goebel	.	.	.	X	.	.	.	X	.	.	.	.	.	.	X	.	X	X	.	.	X	.	X	.	.	16
*Cheilolejeunea trapezia* (Nees) R.M.Schust. & Kachroo	.	.	.	.	.	.	X	X	X	.	.	X	.	X	X	X	X	X	.	.	.	X	.	.	.	15
*Cololejeunea lanciloba* Steph.	X	.	X	X	.	.	.	.	.	.	.	X	X	.	.	.	X	.	.	.	.	.	.	.	.	11
*Cololejeunea longifolia* (Mitt.) Mizut.	.	.	.	X	.	X	.	.	.	.	.	.	.	X	X	.	.	.	X	.	X	.	X	.	.	11
*Leptolejeunea vitrea* (Nees) Schiffn.	.	.	.	.	.	.	.	.	.	X	X	.	.	.	.	.	.	.	.	.	.	.	.	.	.	8
*Microlejeunea punctiformis* Taylor (Steph.)	.	.	.	.	.	.	X	X	.	.	.	.	.	.	X	.	X	.	.	.	.	.	.	.	X	6
*Cololejeunea hildebrandii* (Austin) Steph.	.	.	.	.	.	.	.	.	.	.	X	.	.	.	.	X	.	.	X	.	.	.	.	X	.	5
*Cololejeunea peponiformis* Mizut.	.	.	X	.	.	.	.	.	.	.	.	.	.	.	.	X	X	.	.	.	.	.	.	.	.	5
*Drepanolejeunea pentadactyla* (Mont.) Steph.	.	.	.	.	.	.	.	.	.	.	.	.	.	.	.	.	.	.	.	.	.	.	.	.	.	5
*Lejeunea* sp.2	.	.	.	.	.	.	.	.	.	.	.	.	.	.	.	.	.	.	.	.	.	.	.	.	.	5
*Caudalejeunea reniloba* (Gottsche) Steph.	.	.	.	.	.	.	X	.	.	.	.	.	.	.	.	.	.	.	.	.	.	.	.	.	.	4
*Cololejeunea tenella* Benedix	.	.	.	.	.	.	.	.	.	.	.	.	.	.	.	.	.	.	.	.	.	.	.	.	.	4
*Colura acroloba* (Steph.) Ast	.	.	.	.	.	.	.	.	.	.	.	.	.	.	.	.	.	.	.	.	.	.	.	.	.	4
*Frullania* sp.	.	.	.	.	.	.	.	.	.	.	.	.	.	.	.	.	X	X	.	.	.	.	.	.	.	4
*Lopholejeunea subfusca* (Nees) Schiffn.	.	X	.	.	.	.	.	X	.	.	.	.	.	.	.	.	.	.	.	.	.	.	.	.	.	4
*Cololejeunea acuminata* Mizut.	.	.	.	.	.	.	X	.	.	.	.	.	.	.	X	.	.	.	.	.	.	.	.	X	.	3
*Cololejeunea raduliloba* Steph.	.	.	.	.	.	.	.	.	.	.	.	.	.	.	.	.	.	.	.	.	.	.	.	.	.	3
*Drepanolejeunea vesiculosa* (Mitt.) Steph.	.	.	.	.	.	.	X	X	.	.	.	.	.	.	.	.	.	.	.	.	.	.	.	.	.	3
*Leptolejeunea elliptica* (Lehm. & Lindenb.) Schiffn.	.	.	.	.	.	.	.	.	.	.	.	.	.	.	.	.	.	.	.	.	.	.	.	.	.	3
*Cheilolejeunea* (*Cyrtolejeunea*?)	.	.	.	.	.	.	.	.	.	.	.	.	.	.	.	.	.	.	.	.	.	.	.	.	.	2
*Colura conica* (Sande Lac.) K.I.Goebel	.	.	.	.	.	.	.	.	.	.	.	.	.	.	.	.	.	.	.	.	.	.	.	.	.	2
*Colura corynophora* (Nees et al.) Trevis	.	.	.	.	.	.	.	.	.	.	.	.	.	.	.	.	.	.	.	.	.	.	.	.	.	2
*Leptolejeuna ligulata* Herzog	.	.	.	X	.	.	.	.	.	.	.	.	.	.	.	.	.	.	.	.	.	.	.	.	.	2
*Leptolejeunea tripuncta* (Mitt.) Steph.	.	X	.	.	.	.	.	.	.	.	.	.	.	.	.	.	.	.	.	.	.	.	.	.	.	2
*Cheilolejeunea intertexta* (Lindenb.) Steph.	.	.	.	.	.	.	.	.	.	.	.	.	.	.	.	X	.	.	.	.	.	.	.	.	.	1
*Cheilolejeunea vittata* (G.Hoffm.) R.M.Schust & Kachroo	.	.	.	.	.	.	.	.	.	.	.	.	X	.	.	.	.	.	.	.	.	.	.	.	.	1
Cololejeunea aff. schmidtii Steph.	.	.	.	.	X	.	.	.	.	.	.	.	.	.	.	.	.	.	.	.	.	.	.	.	.	1
*Cololejeunea stylosa* (Steph.) Mizut.	.	.	.	.	.	.	.	.	.	.	.	.	.	.	.	.	.	.	.	.	.	.	.	.	.	1
*Colura superba* (Mont.) Steph.	.	.	.	.	.	.	.	.	.	.	.	.	.	.	.	.	.	.	.	.	.	.	.	.	.	1
*Colura ornata* K.I.Goebel	.	.	.	.	.	.	.	.	.	.	.	.	.	.	.	.	.	.	.	.	.	.	.	.	.	1
*Lejeunea flava* (Sw.) Nees	.	.	.	.	.	.	.	.	.	.	.	.	.	.	.	.	.	.	.	.	.	.	.	.	.	1
*Lopholejeunea nigricans* (Lindenb.) Schiffn.	.	.	.	.	.	.	.	.	.	.	.	.	.	.	X	.	.	.	.	.	.	.	.	.	.	1
*Microlejeunea filicuspis* (Steph.) Heinrichs et al.	.	.	.	.	.	.	.	.	.	.	.	.	.	.	.	.	X	.	.	.	.	.	.	.	.	1

**Table 3.** The epiphyllous communities in Mt Silam, a lower montane rainforest at 600–740 m elevation.

**Leaf surface area in cm^2^**	**33**	**100**	**50**	**63**	**100**	**62**	**42**	**94**	**68**	**25**	**25**	**65**	**100**	**57**	**48**	**26**	**25**	**72**	**130**	**65**	**110**	**52**	**85**	**19**	**21**	
**Cover of epiphylls in % of leaf surface**	**60**	**85**	**35**	**65**	**18**	**16**	**30**	**28**	**30**	**15**	**18**	**38**	**28**	**80**	**35**	**40**	**18**	**8**	**30**	**48**	**12**	**30**	**65**	**45**	**2**	
**Species number of each leaf**	**2**	**4**	**9**	**5**	**8**	**6**	**11**	**7**	**9**	**4**	**8**	**3**	**6**	**5**	**4**	**2**	**3**	**4**	**8**	**3**	**11**	**4**	**6**	**2**	**3**	
*Drepanolejeunea tricornua* Herzog	●	+	+	●	●	.	●	●	●	.	.	●	●	+	●	●	.	+	●	●	●	●	●	●	.	
*Drepanolejeunea pentadactyla* (Mont.) Steph.	.	.	+	.	+	.	+	+	.	.	.	+	+	●	+	+	.	.	●	.	+	.	.	.	.	
Leptolejeunea aff. balansae Steph.	.	.	+	+	+	●	+	.	+	.	.	.	+	.	+	.	.	+	+	.	+	.	.	+	+	
*Cheilolejeunea trapezia* (Nees) R.M.Schust. & Kachroo	.	+	+	+	+	+	+	.	.	.	.	+	●	.	.	.	.	.	+	+	+	+	+	.	.	
*Leptolejeunea amphiophthalma* Zwickel	.	.	+	.	+	.	.	.	.	.	.	.	+	+	.	.	+	.	+	.	+	.	.	.	.	
*Colura corynophora* (Nees et al.) Trevis	.	.	.	.	.	.	.	.	+	.	.	.	.	.	.	.	.	.	.	.	+	.	.	.	.	
*Cololejeunea mutabilis* Benedix	.	.	.	.	.	.	+	+	.	+	+	.	.	.	.	.	.	+	.	.	+	+	+	.	.	
*Colura conica* (Sande Lac.) K.I.Goebel	.	.	.	.	.	+	.	.	.	.	.	.	.	.	.	.	.	.	+	.	.	+	.	.	.	
*Colura* sp.	.	+	+	+	+	.	+	+	.	.	.	.	.	.	.	.	.	.	.	.	.	.	.	.	.	
*Drepanolejeunea dactylophora* (Nees et al.) Schiffn.	+	●	●	+	.	.	.	.	+	+	.	.	.	.	.	.	.	.	.	.	.	.	.	.	.	
*Cololejeunea equialbi* Tixier	.	.	.	.	.	.	.	.	+	+	+	.	.	.	.	.	+	.	.	.	+	.	+	.	.	
*Cololejeunea metzgeriopsis* (K.I.Goebel) Gradst. et al.	.	.	+	.	+	.	.	.	.	.	.	.	.	.	.	.	.	.	+	.	+	.	.	.	.	
*Metalejeunea cucullata* (Reinw. et al.) Grolle	.	.	.	.	+	+	+	+	.	.	.	.	.	.	.	.	.	.	.	.	.	.	.	.	.	
*Colura superba* (Mont.) Steph.	.	.	.	.	.	.	.	.	.	.	.	.	.	.	.	.	.	.	.	.	.	.	.	.	.	
*Cololejeunea stylosa* (Steph.) Mizut.	.	.	.	.	.	.	.	.	.	.	.	.	.	.	.	.	.	.	.	.	+	.	.	.	.	
*Colura cristata* Ast	.	.	.	.	.	+	.	.	+	.	.	.	.	.	.	.	●	.	.	.	.	.	.	.	.	
*Microlejeunea lunulatiloba* Horik.	.	.	.	.	.	.	.	.	.	.	.	.	.	.	.	.	.	.	.	.	.	.	.	.	.	
*Microlejeunea punctiformis* (Taylor) Steph.	.	.	.	.	.	.	+	+	+	.	+	.	.	.	.	.	.	.	.	.	.	.	.	.	.	
*Tuyamaella serratistipa* S.Hatt.	.	.	.	.	.	.	.	.	.	.	.	.	.	.	.	.	.	.	+	.	.	.	.	.	.	
*Cheilolejeunea parvidens* B.M.Thiers	.	.	.	.	.	.	.	.	.	.	.	.	+	.	.	.	.	.	.	+	.	.	+	.	.	
*Lejeunea papilionacea* Prantl.	.	.	.	.	.	.	.	.	.	.	.	.	.	.	.	.	.	+	.	.	.	.	.	.	.	
*Cheilolejeunea ventricosa* (Schiffn.) Xiao L.He	.	.	.	.	.	.	.	.	+	.	.	.	.	+	.	.	.	.	.	.	.	.	.	.	.	
*Cololejeunea papillosa* (K.I.Goebel) Mizut.	.	.	.	.	.	.	.	.	.	.	.	.	.	.	.	.	.	.	.	.	.	.	+	.	+	
*Colura maxima* Ast	.	.	.	.	.	.	.	.	+	.	.	.	.	.	.	.	.	.	.	.	.	.	.	.	.	
*Frullania apiculata* (Reinw. et al.) Dumort.	.	.	.	.	.	.	.	.	.	.	+	.	.	.	.	.	.	.	.	.	.	.	.	.	.	
*Lejeunea exilis* (Reinw. et al.) Grolle	.	.	.	.	.	.	.	.	.	.	+	.	.	.	.	.	.	.	.	.	.	.	.	.	.	
*Lejeunea micholitzii* Grolle	.	.	.	.	.	.	.	.	.	.	+	.	.	.	.	.	.	.	.	.	.	.	.	.	.	
Leptolejeunea aff. punctata Herzog	.	.	.	.	.	.	.	.	.	.	.	.	.	.	.	.	.	.	.	.	.	.	.	.	.	
*Acromastigum bancanum* (Sande Lac.) A.Evans	.	.	.	.	.	.	.	.	.	.	●	.	.	.	.	.	.	.	.	.	.	.	.	.	.	
*Cheilolejeunea ceylanica* (Gottsche) R.M.Schust.	.	.	.	.	.	.	.	.	.	.	+	.	.	.	.	.	.	.	.	.	.	.	.	.	.	
*Cheilolejeunea intertexta* (Lindenb.) Steph.	.	.	.	.	.	.	+	.	.	.	.	.	.	.	.	.	.	.	.	.	.	.	.	.	.	
*Cheilolejeunea meyeniana* (Nees et al.) R.M.Schust. & Kachroo	.	.	.	.	.	.	.	.	.	.	.	.	.	.	+	.	.	.	.	.	.	.	.	.	.	
*Cheilolejeunea occlusa* (Herzog) T.Kodama & N.Kitag.	.	.	.	.	.	.	+	.	.	.	.	.	.	.	.	.	.	.	.	.	.	.	.	.	.	
*Cheilolejeunea trifaria* (Reinw. et al.) Mizut.	.	.	.	.	.	.	.	.	.	.	.	.	.	.	.	.	.	.	.	.	.	.	.	.	.	
*Cheilolejeunea* sp.	.	.	.	.	.	.	.	.	.	.	.	.	.	.	.	.	.	.	.	.	.	.	.	.	.	
*Cololejeunea haskarliana* (Lehm. & Lindenb.) Schiffn.	.	.	.	.	.	.	.	.	.	.	.	.	.	.	.	.	.	.	.	.	.	.	.	.	.	
*Cololejrunea obliqua* (Nees & Mont.) Schiffn.	.	.	.	.	.	.	+	.	.	.	.	.	.	.	.	.	.	.	.	.	.	.	.	.	.	
*Colura acroloba* (Steph.) Ast	.	.	.	.	.	.	.	.	.	.	.	.	.	.	.	.	.	.	.	.	+	.	.	.	.	
Colura aff. mosenii Steph.	.	.	.	.	.	+	.	.	.	.	.	.	.	.	.	.	.	.	.	.	.	.	.	.	.	
*Diplasiolejeunea cavifolia* Steph.	.	.	.	.	.	.	.	.	.	.	.	.	.	.	.	.	.	.	.	.	.	.	.	.	+	
*Diplasiolejeunea* sp.	.	.	.	.	.	.	.	.	.	.	.	.	.	.	.	.	.	.	.	.	.	.	.	.	.	
*Drepanolejeunea longicornua* (Herzog) Mizut.	.	.	●	.	.	.	.	.	.	.	.	.	.	.	.	.	.	.	.	.	.	.	.	.	.	
*Drepanolejeunea serricalyx* Herzog	.	.	.	.	.	.	.	.	.	.	.	.	.	+	.	.	.	.	.	.	.	.	.	.	.	
*Drepanolejeunea ternatensis* (Gottsche) Schiffn.	.	.	.	.	.	.	.	.	.	.	.	.	.	.	.	.	.	.	.	.	.	.	.	.	.	
Lejeunea cf. tuberculosa Steph.	.	.	.	.	.	.	.	.	.	.	.	.	.	.	.	.	.	.	.	.	.	.	.	.	.	
*Lepidolejeunea bidentula* (Steph.) R.M.Schust.	.	.	.	.	.	.	.	.	.	+	.	.	.	.	.	.	.	.	.	.	.	.	.	.	.	
*Leptolejeunea elliptica* (Lehm. & Lindenb.) Schiffn.	.	.	.	.	.	.	.	+	.	.	.	.	.	.	.	.	.	.	.	.	.	.	.	.	.	
**Leaf surface area in cm^2^**	**90**	**52**	**150**	**150**	**55**	**160**	**106**	**120**	**136**	**130**	**45**	**65**	**42**	**110**	**8**	**41**	**16**	**36**	**19**	**155**	**72**	**20**	**15**	**120**	**120**	**50**
**Cover of epiphylls in % of leaf surface**	**25**	**55**	**5**	**5**	**55**	**20**	**15**	**8**	**10**	**45**	**50**	**35**	**70**	**6**	**12**	**40**	**60**	**18**	**15**	**5**	**20**	**40**	**30**	**15**	**5**	
**Species number of each leaf**	**4**	**5**	**6**	**4**	**4**	**7**	**3**	**4**	**5**	**3**	**1**	**4**	**3**	**3**	**4**	**6**	**3**	**4**	**6**	**9**	**4**	**4**	**2**	**4**	**1**	
*Drepanolejeunea tricornua* Herzog	●	+	.	.	●	●	●	.	●	●	●	●	●	.	●	●	●	●	.	.	.	●	●	.	+	37
*Drepanolejeunea pentadactyla* (Mont.) Steph.	+	●	●	.	●	+	●	+	+	.	.	+	+	.	.	+	+	+	.	.	.	+	+	.	.	26
Leptolejeunea aff. balansae Steph.	.	.	.	●	+	.	+	+	+	.	.	.	.	●	.	+	+	+	.	●	●	+	.	●	.	26
*Cheilolejeunea trapezia* (Nees) R.M.Schust. & Kachroo	+	.	+	.	+	.	.	.	+	+	.	+	.	.	.	+	.	.	.	+	.	.	.	.	.	21
*Leptolejeunea amphiophthalma* Zwickel	.	.	.	.	.	+	.	.	.	.	.	.	.	.	.	+	.	.	+	.	.	.	.	.	.	10
*Colura corynophora* (Nees et al.) Trevis	.	+	.	.	.	+	.	●	.	+	.	.	+	.	.	+	.	.	+	.	.	.	.	.	.	9
*Cololejeunea mutabilis* Benedix	.	.	.	.	.	.	.	.	.	.	.	.	.	.	.	.	.	.	.	.	.	.	.	.	.	8
*Colura conica* (Sande Lac.) K.I.Goebel	.	.	.	.	.	.	.	.	.	.	.	.	.	+	.	.	.	.	+	+	.	.	.	+	.	7
*Colura* sp.	.	+	.	.	.	.	.	.	.	.	.	.	.	.	.	.	.	.	.	.	.	.	.	.	.	7
*Drepanolejeunea dactylophora* (Nees et al.) Schiffn.	.	.	.	.	.	.	.	.	.	.	.	.	.	.	.	.	.	.	.	+	.	.	.	.	.	7
*Cololejeunea equialbi* Tixier	.	.	.	.	.	.	.	.	.	.	.	.	.	.	.	.	.	.	.	.	.	.	.	.	.	6
*Cololejeunea metzgeriopsis* (K.I.Goebel) Gradst. et al.	+	.	.	.	.	.	.	.	.	.	.	.	.	.	.	.	.	+	.	.	.	.	.	.	.	6
*Metalejeunea cucullata* (Reinw. et al.) Grolle	.	+	.	.	.	.	.	.	.	.	.	.	.	.	.	.	.	.	.	+	.	.	.	.	.	6
*Colura superba* (Mont.) Steph.	.	.	.	+	.	+	.	+	.	.	.	.	.	.	.	.	.	.	.	+	.	.	.	+	.	5
*Cololejeunea stylosa* (Steph.) Mizut.	.	.	.	+	.	+	.	.	.	.	.	.	.	.	.	.	.	.	.	.	.	.	.	+	.	4
*Colura cristata* Ast	.	.	.	.	.	.	.	.	.	.	.	.	.	.	+	.	.	.	.	.	.	.	.	.	.	4
*Microlejeunea lunulatiloba* Horik.	.	.	+	+	.	.	.	.	.	.	.	+	.	.	.	.	.	.	.	.	+	.	.	.	.	4
*Microlejeunea punctiformis* (Taylor) Steph.	.	.	.	.	.	.	.	.	.	.	.	.	.	.	.	.	.	.	.	.	.	.	.	.	.	4
*Tuyamaella serratistipa* S.Hatt.	.	.	.	.	.	+	.	.	+	.	.	.	.	.	.	.	.	.	.	.	.	+	.	.	.	4
*Cheilolejeunea parvidens* B.M.Thiers	.	.	.	.	.	.	.	.	.	.	.	.	.	.	.	.	.	.	.	.	.	.	.	.	.	3
*Lejeunea papilionacea* Prantl.	.	.	●	.	.	.	.	.	.	.	.	.	.	.	+	.	.	.	.	.	.	.	.	.	.	3
*Cheilolejeunea ventricosa* (Schiffn.) Xiao L.He	.	.	.	.	.	.	.	.	.	.	.	.	.	.	.	.	.	.	.	.	.	.	.	.	.	2
*Cololejeunea papillosa* (K.I.Goebel) Mizut.	.	.	.	.	.	.	.	.	.	.	.	.	.	.	.	.	.	.	.	.	.	.	.	.	.	2
*Colura maxima* Ast	.	.	.	.	.	.	.	.	.	.	.	.	.	.	.	.	.	.	+	.	.	.	.	.	.	2
*Frullania apiculata* (Reinw. et al.) Dumort.	.	.	.	.	.	.	.	.	.	.	.	.	.	.	.	.	.	.	+	.	.	.	.	.	.	2
*Lejeunea exilis* (Reinw. et al.) Grolle	.	.	+	.	.	.	.	.	.	.	.	.	.	.	.	.	.	.	.	.	.	.	.	.	.	2
*Lejeunea micholitzii* Grolle	.	.	.	.	.	.	.	.	.	.	.	.	.	+	.	.	.	.	.	.	.	.	.	.	.	2
Leptolejeunea aff. punctata Herzog	.	.	.	.	.	.	.	.	.	.	.	.	.	.	.	.	.	.	+	+	.	.	.	.	.	2
*Acromastigum bancanum* (Sande Lac.) A.Evans	.	.	.	.	.	.	.	.	.	.	.	.	.	.	.	.	.	.	.	.	.	.	.	.	.	1
*Cheilolejeunea ceylanica* (Gottsche) R.M.Schust.	.	.	.	.	.	.	.	.	.	.	.	.	.	.	.	.	.	.	.	.	.	.	.	.	.	1
*Cheilolejeunea intertexta* (Lindenb.) Steph.	.	.	.	.	.	.	.	.	.	.	.	.	.	.	.	.	.	.	.	.	.	.	.	.	.	1
*Cheilolejeunea meyeniana* (Nees et al.) R.M.Schust. & Kachroo	.	.	.	.	.	.	.	.	.	.	.	.	.	.	.	.	.	.	.	.	.	.	.	.	.	1
*Cheilolejeunea occlusa* (Herzog) T.Kodama & N.Kitag.	.	.	.	.	.	.	.	.	.	.	.	.	.	.	.	.	.	.	.	.	.	.	.	.	.	1
*Cheilolejeunea trifaria* (Reinw. et al.) Mizut.	.	.	●	.	.	.	.	.	.	.	.	.	.	.	.	.	.	.	.	.	.	.	.	.	.	1
*Cheilolejeunea* sp.	.	.	.	.	.	.	.	.	.	.	.	.	.	.	.	.	.	.	.	.	+	.	.	.	.	1
*Cololejeunea haskarliana* (Lehm. & Lindenb.) Schiffn.	.	.	.	.	.	.	.	.	.	.	.	.	.	.	+	.	.	.	.	.	.	.	.	.	.	1
*Cololejrunea obliqua* (Nees & Mont.) Schiffn.	.	.	.	.	.	.	.	.	.	.	.	.	.	.	.	.	.	.	.	.	.	.	.	.	.	1
*Colura acroloba* (Steph.) Ast	.	.	.	.	.	.	.	.	.	.	.	.	.	.	.	.	.	.	.	.	.	.	.	.	.	1
Colura aff. mosenii Steph.	.	.	.	.	.	.	.	.	.	.	.	.	.	.	.	.	.	.	.	.	.	.	.	.	.	1
*Diplasiolejeunea cavifolia* Steph.	.	.	.	.	.	.	.	.	.	.	.	.	.	.	.	.	.	.	.	.	.	.	.	.	.	1
*Diplasiolejeunea* sp.	.	.	.	.	.	.	.	.	.	.	.	.	.	.	.	.	.	.	.	+	.	.	.	.	.	1
*Drepanolejeunea longicornua* (Herzog) Mizut.	.	.	.	.	.	.	.	.	.	.	.	.	.	.	.	.	.	.	.	.	.	.	.	.	.	1
*Drepanolejeunea serricalyx* Herzog	.	.	.	.	.	.	.	.	.	.	.	.	.	.	.	.	.	.	.	.	.	.	.	.	.	1
*Drepanolejeunea ternatensis* (Gottsche) Schiffn.	.	.	.	.	.	.	.	.	.	.	.	.	.	.	.	.	.	.	.	+	.	.	.	.	.	1
Lejeunea cf. tuberculosa Steph.	.	.	.	.	.	.	.	.	.	.	.	.	.	.	.	.	.	.	.	.	+	.	.	.	.	1
*Lepidolejeunea bidentula* (Steph.) R.M.Schust.	.	.	.	.	.	.	.	.	.	.	.	.	.	.	.	.	.	.	.	.	.	.	.	.	.	1
*Leptolejeunea elliptica* (Lehm. & Lindenb.) Schiffn.	.	.	.	.	.	.	.	.	.	.	.	.	.	.	.	.	.	.	.	.	.	.	.	.	.	1

**Table 4.** The epiphyllous communities in Mt. Alab, a mossy cloud (elfin) forest at 1900–1940 m elevation.

**Leaf surface area in cm^2^**	**100**	**75**	**72**	**100**	**60**	**60**	**70**	**35**	**80**	**63**	**36**	**100**	**35**	**16**	**67**	**22**	**27**	**50**	**15**	**60**	**36**	**207**	**41**	**26**	**120**	
**Cover of epiphylls in % of leaf surface**	**30**	**12**	**30**	**28**	**15**	**40**	**70**	**40**	**20**	**25**	**30**	**8**	**30**	**45**	**8**	**15**	**18**	**14**	**55**	**30**	**30**	**25**	**65**	**65**	**2**	
**Species number of each leaf**	**10**	**7**	**5**	**8**	**10**	**8**	**70**	**4**	**6**	**11**	**7**	**9**	**6**	**7**	**4**	**4**	**5**	**7**	**8**	**8**	**4**	**13**	**7**	**9**	**4**	
*Drepanolejeuna thwaitesiana* (Mitt.) Steph.	X	X	.	.	X	X	X	X	.	X	X	X	X	.	X	.	X	X	X	.	.	X	X	.	.	
*Diplasiolejeunea jovet-astiae* Grolle	X	X	.	X	.	X	.	.	X	X	.	X	.	X	.	.	X	.	X	.	.	X	.	.	.	
*Drepanolejeunea dactylophora* (Nees et al.) Schiffn.	.	.	.	X	.	.	X	.	.	X	X	X	X	.	X	X	X	.	X	X	.	.	X	.	X	
*Cololejeunea peraffinis* (Schiffn.) Schiffn.	.	X	.	.	X	X	X	.	.	X	X	.	X	X	.	X	.	X	X	X	.	X	X	X	X	
*Leptolejeunea elliptica* (Lehm. & Lindenb.) Schiffn.	.	X	X	.	X	.	X	X	X	.	.	.	.	.	.	.	.	X	.	X	.	.	.	.	X	
*Drepanolejeunea pentadactyla* (Mont.) Steph.	X	.	X	X	.	.	X	X	.	.	X	X	.	.	.	.	.	.	X	.	X	X	.	.	.	
*Microlejeunea punctiformis* (Taylor) Steph.	X	.	.	.	X	.	.	.	X	X	.	X	.	.	.	.	.	.	.	X	.	X	.	X	.	
*Drepanolejeunea tenera* K.I.Goebel	.	.	.	.	X	.	.	.	X	.	X	.	.	.	.	.	X	.	.	X	.	.	.	X	.	
*Cololejeunea haskarliana* (Lehm. & Lindenb.) Schiffn.	.	.	X	.	.	.	X	.	.	.	X	.	.	.	.	X	.	.	X	.	X	.	X	X	.	
*Drepanolejeunea vesiculosa* (Mitt.) Steph.	X	.	.	X	.	X	.	.	.	.	.	X	X	X	.	.	.	.	X	.	.	.	.	.	.	
*Cololejeunea ensifera* Tixier	.	X	.	.	.	.	.	.	.	X	.	.	X	.	.	X	.	.	.	.	.	.	X	.	X	
*Leptolejeunea subdentata* Herzog	.	.	X	.	X	X	.	.	X	.	.	.	.	.	.	.	.	.	.	.	.	.	.	.	.	
*Frullania ramuligera* (Nees) Mont.	X	.	.	X	.	.	.	.	.	.	.	X	.	X	.	.	.	X	.	.	.	X	.	.	.	
*Cololejeunea dozyana* (Sande Lac.) Schiffn.	.	.	.	X	.	X	.	.	.	X	.	.	.	.	.	.	.	.	.	.	.	X	.	.	.	
*Cololejeunea macounii* (Underw.) A.Evans	.	.	.	.	X	.	.	.	.	.	.	.	.	.	X	.	X	.	.	.	.	.	.	.	.	
*Cololejeunea stephanii* Benedix	.	X	X	.	.	.	.	.	X	.	.	.	.	.	X	.	.	.	.	.	.	.	X	.	.	
*Cheilolejeunea trapezia* (Nees et al.) R.M.Schust. & Kachroo	.	.	.	.	.	.	.	.	.	.	.	.	.	.	.	.	.	X	.	.	.	X	.	X	.	
*Colura tenuicornis* (A.Evans) Steph.	.	.	.	X	X	.	.	X	.	.	.	.	.	.	.	.	.	X	.	.	.	.	.	.	.	
*Lejeunea flava* (Sw.) Nees	X	.	.	.	X	.	.	.	.	.	.	.	.	X	.	.	.	.	.	.	.	.	.	.	.	
*Cololejeunea papillosa* (K.I.Goebel) Mizut.	.	.	.	.	.	X	.	.	.	.	.	.	.	.	.	.	.	.	.	.	.	X	.	X	.	
*Cololejeunea sphaerodonta* Mizut.	.	.	.	.	.	.	X	.	.	.	X	.	.	.	.	.	.	.	X	.	X	X	.	.	.	
*Colura verdornii* Herzog & Ast	.	X	.	.	X	X	.	.	.	.	.	.	.	.	.	.	.	X	.	.	.	X	.	.	.	
Drepanolejeunea aff. serricalyx Herzog	.	.	.	.	.	.	.	.	.	.	.	X	X	.	.	.	.	.	.	X	.	.	X	.	.	
*Lejeunea* sp.	X	.	.	.	.	.	.	.	.	.	.	.	.	.	.	.	.	.	.	.	.	X	.	X	.	
*Leptolejeunea maculata* (Mitt.) Schiffn.	.	.	.	X	.	.	.	.	.	.	.	.	.	.	.	.	.	.	.	.	.	X	.	.	.	
*Frullania* sp.	X	.	.	.	.	.	.	.	.	.	.	.	.	X	.	.	.	.	.	.	.	.	.	.	.	
*Drepanolejeunea fissicornua* Steph.	.	.	.	.	.	.	.	.	.	.	.	.	.	.	.	.	.	.	.	.	.	.	.	.	.	
*Microlejeunea constricta* (Grolle) Grolle	X	.	.	.	.	.	.	.	.	.	.	.	.	.	.	.	.	.	.	X	.	.	.	.	.	
*Radula tjibodensis* K.I.Goebel	.	.	.	.	.	.	.	.	.	.	.	.	.	.	.	.	.	.	.	.	X	.	.	.	.	
*Schiffneriolejeunea tumida* (Nees) Gradst.	.	.	.	.	.	.	.	.	.	X	.	.	.	.	.	.	.	.	.	.	.	.	.	.	.	
Cheilolejeunea aff. ventricosa (Schiffn.) Xiao L.He	.	.	.	.	.	.	.	.	.	.	.	.	.	.	.	.	.	.	.	X	.	.	.	.	.	
*Cheilolejeunea occlusa* (Herzog) T.Kodama & N.Kitag.	.	.	.	.	.	.	.	.	.	.	.	X	.	.	.	.	.	.	.	.	.	.	.	.	.	
*Cheilolejeunea meyeniana* (Nees et al.) R.M.Schust. & Kachroo	.	.	.	.	.	.	.	.	.	.	.	.	.	X	.	.	.	.	.	.	.	.	.	.	.	
Cololejeunea cf. filicaulis Steph.	.	.	.	.	.	.	.	.	.	.	.	.	.	.	.	.	.	.	.	.	.	.	.	X	.	
*Cololejeunea magnilobula* (Horik.) S.Hatt.	.	.	.	.	.	.	.	.	.	X	.	.	.	.	.	.	.	.	.	.	.	.	.	.	.	
*Cololejeunea* sp.	.	.	.	.	.	.	.	.	.	.	.	.	.	.	.	.	.	.	.	.	.	.	.	.	.	
*Colura* sp.	.	.	.	.	.	.	.	.	.	.	.	.	.	.	.	.	.	.	.	.	.	.	.	.	.	
*Drepanolejeunea teysmannii* Steph.	.	.	.	.	.	.	.	.	.	X	.	.	.	.	.	.	.	.	.	.	.	.	.	.	.	
*Drepanolejeunea ternatensis* (Gottsche) Schiffn.	.	.	.	.	.	.	.	.	.	.	.	.	.	.	.	.	.	.	.	.	.	.	.	.	.	
*Lopholejeunea eulopha* (Taylor) Schiffn.	.	.	.	.	.	.	.	.	.	.	.	.	.	.	.	.	.	.	.	.	.	.	.	X	.	
*Metalejeunea cucullata* (Reinw. et al.) Grolle	.	.	.	.	.	.	.	.	.	.	.	.	.	.	.	.	.	.	.	.	.	.	.	.	.	
*Myriocoleopsis minutissima* (Sm.) R.L.Zhu et al.	.	.	.	.	.	.	.	.	.	X	.	.	.	.	.	.	.	.	.	.	.	.	.	.	.	
**Leaf surface area in cm^2^**	**20**	**40**	**13**	**30**	**54**	**20**	**26**	**12**	**5**	**10**	**13**	**43**	**20**	**50**	**145**	**18**	**110**	**65**	**13**	**20**	**60**	**86**	**60**	**93**	**20**	**FR**
**Cover of epiphylls in % of leaf surface**	**20**	**65**	**60**	**16**	**2**	**20**	**19**	**25**	**60**	**5**	**50**	**10**	**25**	**45**	**8**	**25**	**8**	**14**	**40**	**15**	**9**	**12**	**12**	**9**	**60**	**A**
**Species number of each leaf**	**5**	**3**	**6**	**5**	**4**	**5**	**4**	**5**	**6**	**5**	**5**	**6**	**6**	**7**	**5**	**7**	**9**	**9**	**6**	**7**	**8**	**6**	**7**	**9**	**4**	**50**
*Drepanolejeuna thwaitesiana* (Mitt.) Steph.	X	X	X	X	X	X	.	X	.	X	.	X	.	X	X	X	X	.	.	.	X	.	X	X	.	32
*Diplasiolejeunea jovet-astiae* Grolle	X	.	X	X	.	X	X	.	X	X	X	.	.	.	.	X	X	X	X	X	X	.	X	X	.	26
*Drepanolejeunea dactylophora* (Nees et al.) Schiffn.	X	.	X	X	X	.	X	X	X	.	X	X	X	X	.	X	.	.	.	.	.	.	.	X	.	26
*Cololejeunea peraffinis* (Schiffn.) Schiffn.	.	.	.	.	.	.	.	X	.	.	.	.	X	X	.	.	X	.	.	.	.	.	X	X	X	22
*Leptolejeunea elliptica* (Lehm. & Lindenb.) Schiffn.	X	.	X	.	.	X	.	.	X	.	.	.	.	.	X	X	X	X	.	.	X	X	.	X	.	21
*Drepanolejeunea pentadactyla* (Mont.) Steph.	.	X	.	.	.	.	.	.	.	X	.	.	.	X	.	.	.	X	X	.	.	X	X	X	.	18
*Microlejeunea punctiformis* (Taylor) Steph.	.	.	.	.	.	.	.	.	.	.	X	X	.	.	.	X	.	X	X	X	X	.	.	X	.	16
*Drepanolejeunea tenera* K.I.Goebel	.	.	.	.	.	.	.	.	.	.	.	.	.	.	X	X	X	X	X	X	X	.	.	.	X	13
*Cololejeunea haskarliana* (Lehm. & Lindenb.) Schiffn.	.	.	X	X	.	.	.	.	X	.	.	.	.	X	.	.	.	.	.	.	.	.	.	.	.	12
*Drepanolejeunea vesiculosa* (Mitt.) Steph.	.	.	.	.	.	.	.	.	.	.	X	X	.	.	.	.	.	X	X	.	.	.	.	X	.	12
*Cololejeunea ensifera* Tixier	.	.	.	.	.	.	.	.	X	.	X	.	X	.	.	.	.	X	.	X	.	.	.	.	.	11
*Leptolejeunea subdentata* Herzog	.	.	.	.	.	X	.	.	.	.	.	.	.	.	X	X	.	.	.	.	X	X	X	.	.	10
*Frullania ramuligera* (Nees) Mont.	.	.	.	.	.	.	.	.	.	.	.	.	X	.	.	.	X	.	.	X	.	.	.	.	.	9
*Cololejeunea dozyana* (Sande Lac.) Schiffn.	.	.	X	.	.	.	X	.	.	.	.	.	.	.	.	.	.	.	.	.	X	.	X	.	X	8
*Cololejeunea macounii* (Underw.) A.Evans	.	X	.	X	.	.	.	.	.	X	.	.	.	.	.	.	.	.	.	.	.	X	X	.	.	8
*Cololejeunea stephanii* Benedix	.	.	.	.	.	.	.	.	.	.	.	X	.	.	.	.	.	.	.	.	.	X	.	.	.	8
*Cheilolejeunea trapezia* (Nees et al.) R.M.Schust. & Kachroo	.	.	.	.	.	.	X	.	.	.	.	.	.	.	.	.	X	.	.	.	X	.	.	X	.	7
*Colura tenuicornis* (A.Evans) Steph.	X	.	.	.	.	X	.	X	.	.	.	.	.	.	.	.	.	.	.	.	.	.	.	.	.	7
*Lejeunea flava* (Sw.) Nees	.	.	.	.	X	.	.	.	.	.	.	.	X	.	.	.	.	.	X	.	.	.	.	.	.	6
*Cololejeunea papillosa* (K.I.Goebel) Mizut.	.	.	.	.	.	.	.	.	.	.	.	.	.	X	X	.	.	.	.	.	.	.	.	.	.	5
*Cololejeunea sphaerodonta* Mizut.	.	.	.	.	.	.	.	.	.	.	.	.	.	.	.	.	.	.	.	.	.	.	.	.	.	5
*Colura verdornii* Herzog & Ast	.	.	.	.	.	.	.	.	.	.	.	.	.	.	.	.	.	.	.	.	.	.	.	.	.	5
Drepanolejeunea aff. serricalyx Herzog	.	.	.	.	.	.	.	.	.	.	.	X	.	.	.	.	.	.	.	.	.	.	.	.	.	5
*Lejeunea* sp.	.	.	.	.	.	.	.	.	.	.	.	.	.	.	.	.	X	X	.	.	.	.	.	.	.	5
*Leptolejeunea maculata* (Mitt.) Schiffn.	.	.	.	.	.	.	.	X	.	.	.	.	.	.	.	.	.	.	.	.	.	X	.	.	.	4
*Frullania* sp.	.	.	.	.	.	.	.	.	.	.	.	.	.	.	.	.	X	.	.	.	.	.	.	.	.	3
*Drepanolejeunea fissicornua* Steph.	.	.	.	.	.	.	.	.	X	X	.	.	.	.	.	.	.	.	.	.	.	.	.	.	X	2
*Microlejeunea constricta* (Grolle) Grolle	.	.	.	.	.	.	.	.	.	.	.	.	.	.	.	.	.	.	.	.	.	.	.	.	.	2
*Radula tjibodensis* K.I.Goebel	.	.	.	.	.	.	.	.	.	.	.	.	.	X	.	.	.	.	.	.	.	.	.	.	.	2
*Schiffneriolejeunea tumida* (Nees) Gradst.	.	.	.	.	.	.	.	.	.	.	.	.	.	.	.	.	.	.	.	X	.	.	.	.	.	2
Cheilolejeunea aff. ventricosa (Schiffn.) Xiao L.He	.	.	.	.	.	.	.	.	.	.	.	.	.	.	.	.	.	.	.	.	.	.	.	.	.	1
*Cheilolejeunea occlusa* (Herzog) T.Kodama & N.Kitag.	.	.	.	.	.	.	.	.	.	.	.	.	.	.	.	.	.	.	.	.	.	.	.	.	.	1
*Cheilolejeunea meyeniana* (Nees et al.) R.M.Schust. & Kachroo	.	.	.	.	.	.	.	.	.	.	.	.	.	.	.	.	.	.	.	.	.	.	.	.	.	1
Cololejeunea cf. filicaulis Steph.	.	.	.	.	.	.	.	.	.	.	.	.	.	.	.	.	.	.	.	.	.	.	.	.	.	1
*Cololejeunea magnilobula* (Horik.) S.Hatt.	.	.	.	.	.	.	.	.	.	.	.	.	.	.	.	.	.	.	.	.	.	.	.	.	.	1
*Cololejeunea* sp.	.	.	.	.	X	.	.	.	.	.	.	.	.	.	.	.	.	.	.	.	.	.	.	.	.	1
*Colura* sp.	.	.	.	.	.	.	.	.	.	.	.	.	.	.	.	.	.	X	.	.	.	.	.	.	.	1
*Drepanolejeunea teysmannii* Steph.	.	.	.	.	.	.	.	.	.	.	.	.	.	.	.	.	.	.	.	.	.	.	.	.	.	1
*Drepanolejeunea ternatensis* (Gottsche) Schiffn.	.	.	.	.	.	.	.	.	.	.	.	.	.	.	.	.	.	.	.	X	.	.	.	.	.	1
*Lopholejeunea eulopha* (Taylor) Schiffn.	.	.	.	.	.	.	.	.	.	.	.	.	.	.	.	.	.	.	.	.	.	.	.	.	.	1
*Metalejeunea cucullata* (Reinw. et al.) Grolle	.	.	.	.	.	.	.	.	.	.	.	.	X	.	.	.	.	.	.	.	.	.	.	.	.	1
*Myriocoleopsis minutissima* (Sm.) R.L.Zhu et al.	.	.	.	.	.	.	.	.	.	.	.	.	.	.	.	.	.	.	.	.	.	.	.	.	.	1

**Table 5.** The comparison of the epiphyllous assemblages of three localities in terms of the number of occurrences of constituting liverwort species.

Locality	Ulu Senagang	Mt. Silam	Mt. Alab	Total
*Leptolejeunea maculata* (Mitt.) Schiffn.	32	26	4	62
*Drepanolejeunea pentadactyla* (Mont.) Steph.	5	24	18	47
*Cheilolejeunea trapezia* (Nees et al.) R.M.Schust. & Kachroo	15	21	7	43
*Microlejeunea punctiformis* (Taylor) Steph.	6	4	16	26
*Leptolejeunea elliptica* (Lehm. & Lindenb.) Schiffn.	3	1	21	25
*Leptolejeunea epiphylla* (Mitt.) Steph.	32	.	.	32
*Cololejeunea planissima* (Mitt.) Abeyw.	22	.	.	22
*Cololejeunea gottschei* (Steph.) Mizut.	19	.	.	19
*Cololejeunea lanciloba* Steph.	11	.	.	11
*Cololejeunea longifolia* (Mitt.) Mizut.	11	.	.	11
*Leptolejeunea vitrea* (Nees) Schiffn.	8	.	.	8
*Cololejeunea hildebrandii* (Austin) Steph.	5	.	.	5
*Cololejeunea peponiformis* Mizut.	5	.	.	5
*Colura corynophora* (Nees et al.) Trevis	2	9	.	11
*Colura conica* (Sande Lac.) K.I.Goebel	2	6	.	9
*Lejeunea* sp. 2	5	1	.	6
*Colura acroloba* (Steph.) Ast	4	1	.	5
*Colura superba* (Mont.) Steph.	1	4	.	5
*Drepanolejeunea tricornua* Herzog	.	37	.	37
*Leptolejeunea amphiophthalma* Zwickel	.	11	.	11
*Cololejeunea mutabilis* Benedix	.	8	.	8
*Colura* sp.	.	7	.	7
*Cololejeunea equialbi* Tixier	.	6	.	6
*Cololejeunea metzgeriopsis* (K.I.Goebel) Gradst. et al.	.	6	.	6
*Colura superba* (Mont.) Steph.	.	5	.	5
*Drepanolejeunea tenera* K.I.Goebel	16	.	13	29
*Lejeunea* sp.	18	.	5	23
*Drepanolejeunea vesiculosa* (Mitt.) Steph.	3	.	12	15
*Frullania* sp.	4	.	3	7
*Lejeunea flava* (Sw.) Nees	1	.	6	7
*Drepanolejeunea dactylophora* (Nees et al.) Schiffn.	.	7	26	35
*Cololejeunea haskarliana* (Lehm. & Lindenb.) Schiffn.	.	1	12	13
*Metalejeunea cucullata* (Reinw. et al.) Grolle	.	6	1	7
*Cololejeunea papillosa* (K.I.Goebel) Mizut.	.	2	5	7
*Cheilolejeunea occlusa* (Herzog) T.Kodama & N.Kitag.	.	1	1	2
*Drepanolejeuna thwaitesiana* (Mitt.) Steph.	.	.	32	32
*Diplasiolejeunea jovet-astiae* Grolle	.	.	26	26
*Cololejeunea peraffinis* (Schiffn.) Schiffn.	.	.	22	22
*Cololejeunea ensifera* Tixier	.	.	11	11
*Leptolejeunea subdentata* Herzog	.	.	10	10
*Frullania ramuligera* (Nees) Mont.	.	.	9	9
*Cololejeunea dozyana* (Sande Lac.) Schiffn.	.	.	8	8
*Cololejeunea macounii* (Underw.) A.Evans	.	.	8	8
*Cololejeunea stephanii* Benedix	.	.	8	8
*Colura tenuicornis* (A.Evans) Steph.	.	.	7	7
*Cololejeunea sphaerodonta* Mizut.	.	.	5	5
*Colura verdoornii* Herzog & Ast	.	.	5	5
Drepanolejeunea aff. serricalyx Herzog	.	.	5	5

**Figure 3.**
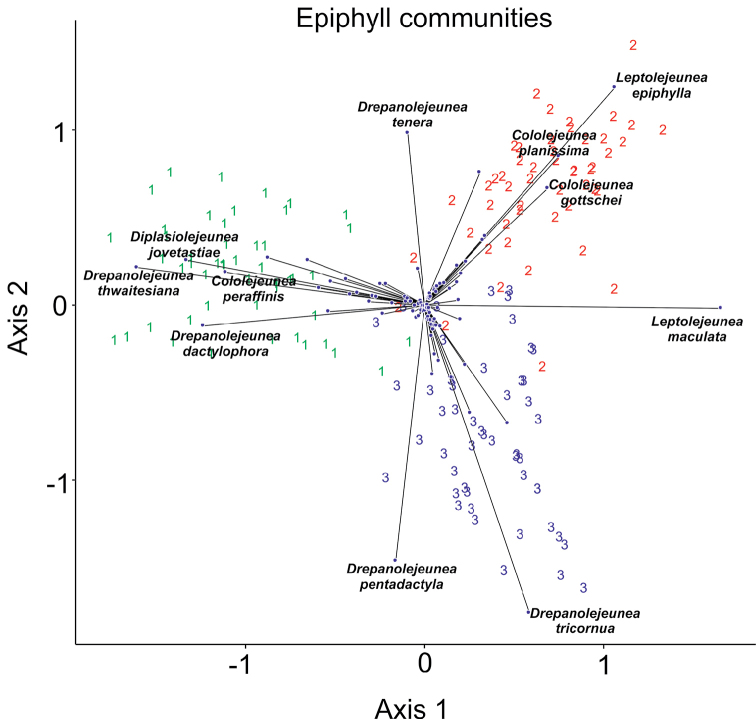
The Principal Components ordination biplot of the three groups of epiphyllous assemblages, each containing 50 leaves. Numbers identify forest sites **1** mossy cloud (elfin) forest, Mt. Alab (Table 4) **2** lowland rainforest, Ulu Senagang (Table 2) **3** lower montane rainforest, Mt. Silam (Table 3).

The entire data set was evaluated by centered principal component analysis. The first two ordination axes explain 14% and 10% of the total variance. Although these percentages may appear low at first sight, the biplot diagram for axes 1–2 (Fig. 3) is well-interpretable. The leaves from the three sites form separate clusters, oriented away from the origin in three directions. The three sites do not separate completely, the species-poor leaves are positioned around the centroid. The length and position of arrows indicate species that are most responsible for the differences between the three sites. It is seen that site number 1 in Fig. 3, i.e. Mt. Alab has a fairly large number of species that typically occur there, such as *Diplasiolejeunea jovet*-*astiae* Grolle, *Drepanolejeunea thwaitesiana* (Mitt.) Steph., *D. dactylophora* (Nees, Lindenb. & Gottsche) Schiffn. and *Cololejeunea peraffinis* (Schiffn.) Schiffn. Site 2 in Ulu Senagang is mostly characterized by the presence of *Leptolejeunea epiphylla* (Mitt.) Steph., *Cololejeunea gottschei* (Steph.) Mizut. and *C. planissima* (Mitt.) Abeyw., whereas *Drepanolejeunea tenera* K.I.Goebel occurs in both sites. In site 3 (Mt. Silam, a lower montane rainforest near to the sea, exposed to rain carrying winds), *Drepanolejeunea pentadactyla* (Mont.) Steph. and *D. tricornua* Herzog appear most typical. Most species are positioned near the origin, showing that they are either relatively rare as *Cololejeunea macounii* (Underw.) A.Evans or *Colura superba* (Mont.) Steph. or common to all the three sites like *Leptolejeunea maculata* (Mitt.) Schiffn.

**Figure 4.**
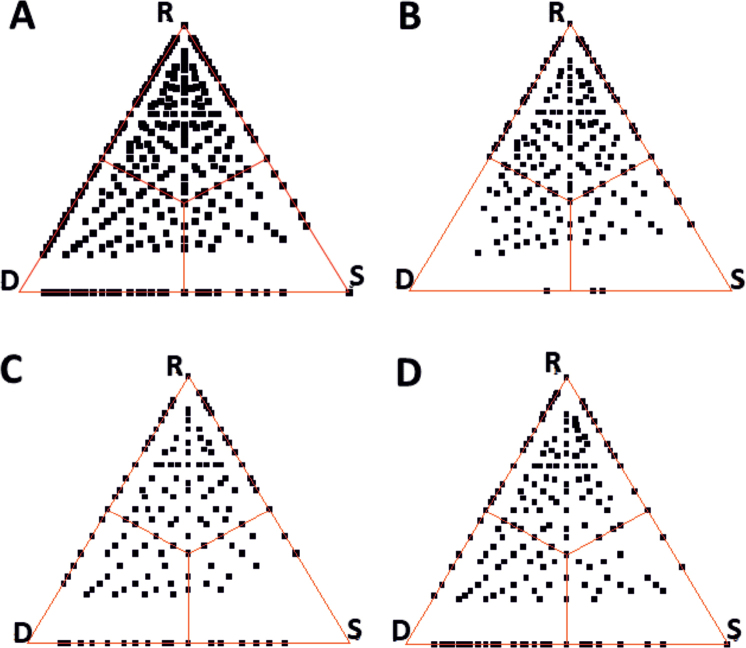
Ternary (or simplex) plot for the epiphyllous liverwort assemblages based on presence-absence data for three rainforest sites in Sabah **A** all sites taken together **B** mossy cloud (elfin) forest, Mt. Alab **C** lowland rainforest, Ulu Senagang **D** lower montane rainforest, Mt. Silam.

The SDR simplex plot and associated percentages obtained for the entire study area (three sites taken together) demonstrate that there is an extremely high beta diversity (91%) of epiphyllous assemblages in the study sites, leaving only a 9% share by similarity (Fig. 4A). Beta diversity is dominated by turnover (species replacement, R = 66%) while richness difference (D) is 25%. Its graphical manifestation is that most of the points (each representing a pair of leaves) lie within or near the upper third of the triangle (R – replacement). The anti-nestedness fraction within beta diversity, corresponding to points lying on the left edge of the triangle, is 11% – this is caused by pairs of leaves that do not have a single species in common. Nevertheless, quite many points lie on the bottom side, demonstrating that nestedness is also characteristic of the epiphyllous bryophyte assemblages – the species occurring in certain leaves are subsets of the species assemblage of other leaves (D + S – Anti-nestedness fraction = 22.5%). The three simplex diagrams obtained for the three forests (Fig. 4B–D) show that the very high overall beta diversity is not merely the result of between-site differences; their beta diversity is 81%, 80% and 80.5%, leaving 19–20% for the similarity component. That is, the liverwort assemblages on the leaves of rainforest trees are extremely diverse. A major difference between the sites is in the partitioning of beta in which species replacement is the highest in the cloud forest, i.e. in Mt. Alab (60%), and the lowest in the lowland montane forest in Mt. Silam (50.5%). This explains why nestedness is much less conspicuous in the cloud forest than elsewhere in which only a few points fall onto the bottom side of the plot.

## Conclusion

The overall conclusion is that the major component of pattern formation in epiphyllous liverwort assemblages from Sabah is species replacement (50–60% for individual forests, 66% for combined data), while richness difference is less pronounced (20–25%). This is in contrast to the results of a study performed on similar assemblages in southern Thailand (Pócs and Podani 2015), where differences in species number were much more influential than species replacement (50% versus 37%). In any case, beta diversity – the sum of richness difference and species replacement – is extremely high in both studies, leaving only 10–20% similarity in the species composition of leaf surfaces. The ecological explanation is that the assemblage of a given leaf is likely to be formed by a random choice from the liverwort species pool of the forest, species follow one another haphazardly as allowed by the size of the leaf. In Sabah, the three forests selected for the present study were floristically very different, forming three clusters in the PCA ordination plane with a couple of characteristic species in each. Their separation was not sharp at all, species-poor leaves were arranged around the centroid regardless of their origin, and three sites were overlapping. Extended studies involving more forests from Malaysia, as well as from other areas in south-eastern Asia, may give further insight into the structure of this special type of plant communities.

## References

[B1] Akiyama H Yamaguchi T Suleiman M (2001) The bryophyte flora of Kinabalu National Park (Sabah, Malaysia) based on the collections by Japan-Malaysia collaborative expeditions in 1997. Nature and Human Activities 6: 83–99.

[B2] Anderson MJ Crist TO Chase JM Vellend M Inouye BD Freestone AL Sanders NJ Cornell HV Comita LS Davies KF Harrison SP Kraft NJB Stegen JC Swenson NG (2011) Navigating the multiple meanings of ß diversity: A roadmap for the practicing ecologist. Ecology Letters 14(1): 19–21. 10.1111/j.1461-0248.2010.01552.x 21070562

[B3] Andi MAM Anuar M Suleiman M (2015) Mosses of Sinua at eastern part of Trusmadi Forest Reserve, Sabah, Malaysia. Sepilok Bulletin 21 & 22: 27–48.

[B4] Bolnick DI Yang LH Fordyce JA Davis JM Svanback R (2002) Measuring individual-level resources specialization. Ecology 83: 2936–2941. 10.1890/0012-9658(2002)083[2936:MILRS]2.0.CO;2

[B5] Bruijnzeel LA Waterloo MJ Proctor JKAT Kuiters AT Kotterink B (1993) Hydrological observations in montane rain forests on Gunung Silam, Sabah, Malaysia, with special reference to the ‘Massenerhebung’ effect. Journal of Ecology 81(1): 145–167. 10.2307/2261231

[B6] Chase JM (2010) Stochastic community assembly causes higher biodiversity in more productive environments. Science 328(5984): 1388–1391. 10.1126/science.1187820 20508088

[B7] Chen PC Wu PC (1964) Study on epiphyllous liverworts of China (I). Zhiwu Fenlei Xuebao 9: 213–276.

[B8] Dinor J Nor Azizi Z Rozi A Aminuddin AG (2007) Deforestation Effect to the Runoff Hydrograph at Sungai Padas Catchment. 2^nd^ International Conference on Managing Rivers in the 21^st^ Century: Solutions Towards Sustainable River Basins. Universiti Sains Malaysia, 351–359.

[B9] Frahm JP Frey W Kürschner H Menzel M (1990) Mosses and liverworts of Mt. Kinabalu. Sabah Parks Publication 12: 1–91.

[B10] Frego KA (2007) Bryophytes as potential indicators of forest integrity. Forest Ecology and Management 242(1): 65–75. 10.1016/j.foreco.2007.01.030

[B11] Gehrig-Downie C Obregon A Benedix J (2013) Diversity and vertical distribution of epiphytic liverworts in lowland rain forest and lowland cloud forest of French Guiana. Journal of Bryology 35(4): 243–254. 10.1179/1743282013Y.0000000070

[B12] Gignac L (2001) Bryophytes as indicators of climate change. The Bryologist 104(3): 410–420. 10.1639/0007-2745(2001)104[0410:BAIOCC]2.0.CO;2

[B13] Goebel KI (1890) Morphologische und biologische Studien. IV. Ueber Javanische Lebermoose. Annales du Jardin Botanique de Buitenzorg 9(1): 1–40.

[B14] Gradstein SR (1997) The taxonomic diversity of epiphyllous bryophytes. Abstracta Botanica 21: 15–19.

[B15] Harrison SP Davies KF Safford HD Viers JH (2006) Beta diversity and the scale-dependence of the productivity-diversity relationship: A test in the Californian serpentine flora. Journal of Ecology 94(1): 110–117. 10.1111/j.1365-2745.2005.01078.x

[B16] Hylander K Nemomissa S Enkosa W (2013) Edge effects on understory epiphytic ferns and epiphyllous bryophytes in moist afromontane forests of Ethiopia. Polish Botanical Journal 58(2): 555–563. 10.2478/pbj-2013-0050

[B17] Hutchison CS (2005) Geology of North-West Borneo, Sarawak, Brunei and Sabah. Elsevier, Amsterdam.

[B18] Inoue H (1989) The bryophytes of Sabah (North Borneo) with special reference to the BRYOTROP transect of Mount Kinabalu. V. *Plagiochila* (Plagiochilaceae, Hepaticae). Willdenowia 18: 555–567.

[B19] Kraft NJB Comita LS Chase JM Sanders NJ Swenson NG Christ TO Stegen JC Vellend M Boyle B Anderson MJ Cornell VH Davies KF Freestone AL Inouye BD Harrison SP Myers JA (2011) Disentangling the drivers of ß diversity along latitudinal and elevational gradients. Science 333(6050): 1755–1758. 10.1126/science.1208584 21940897

[B20] Kraichak E (2014) Microclimate fluctuation correlated with beta diversity of epiphyllous bryophytes communities. Biotropica 46(5): 575–582. 10.1111/btp.12140

[B21] Lücking A (1995) Diversität und Mikrohabitatpräferenzen epiphyller Moose in einem tropischen Regenwald in Costa Rica. Dissertation zur Erlangung des Doktorgrades Dr. rer. nat. der Fakultät für Naturwissenschaften der Universität Ulm, 211 pp.

[B22] Majit HM Suleiman M Rimi R (2011) Diversity and abundance of orchids in Crocker Range Park, Sabah, Malaysia. Journal of Tropical Biology and Conservation 8: 73–81.

[B23] Majuakim L Anthony F (2016) A Note on *Selliguea murudensis* (C. Chr.) Parris (Polypodiaceae), a New Record of Fern for Mount Alab, Crocker Range Park, Sabah. Journal of Tropical Biology & Conservation 13: 119–123.

[B24] Malombe I Matheka KW Pócs T Patino J (2016) Edge effect on epiphyllous bryophytes in Taita Hills fragmented afromontane forests. Journal of Bryology 38(1): 33–46. 10.1179/1743282015Y.0000000015

[B25] Mizutani M (1974) Lepidoziaceae, subfamily Lepidozioideae from Sabah (North Borneo). The Journal of the Hattori Botanical Laboratory 38: 371–385.

[B26] Monge-Najera J (1989) The relationship of epiphyllous liverworts with leaf characteristics and light in Monte Verde. Cryptogamie. Bryologie, Lichenologie 10: 345–352.

[B27] Myers JA Chase JM Jimenez I Jorgensen PM Araujo-Murakami A Paniagua-Zambrana N Seidel R Cornell H (2013) Beta diversity in temperate and tropical forests reflects dissimilar mechanisms of community assembly. Ecology Letters 16(2): 151–157. 10.1111/ele.12021 23113954

[B28] Philippot L Hallin S (2011) Towards food, feed and energy crops mitigating climate change. Trends in Plant Science 16(9): 476–480. 10.1016/j.tplants.2011.05.007 21700487

[B29] Piippo S (1989) The bryophytes of Sabah (North Borneo) with special reference to the BRYOTROP transect of Mount Kinabalu. III. Geocalycaceae (Hepaticae). Willdenowia 18: 513–527.

[B30] Pócs T (1978) Epiphyllous communities and their distribution in East Africa. In: Suire C (Ed.) Congres International de Bryologie, Bordeaux, 21–23 Novembre 1977, Comptes Rendus.Bryophytorum Bibliotheca 13: 681–714.

[B31] Pócs T (1996) Epiphyllous liverworts diversity at worldwide level and its threat and conservation. Anales del Instituto de Biología de la Universidad Nacional Autónoma de México Seris Botanica 67: 109–127.

[B32] Pócs T Podani J (2015) Southern Thailand Bryophytes II. Epiphylls from the Phang-Nga area. Acta Botanica Hungarica 57(1–2): 183–198. 10.1556/ABot.57.2015.1-2.14

[B33] Pócs T Tothmeresz B (1997) Foliicolous bryophyte diversity in tropical rainforests. In: Farkas E Pócs T (Eds) Cryptogams in the Phyllosphaere: Systematics, Distribution, Ecology and Use. Proceedings of the IAB & IAL Symposium on Foliicolous Cryptogams, 29 August – 2 September 1995, Eger, Hungary.Abstracta Botanica 21: 135–144.

[B34] Podani J (2001) SYN-TAX 2000. User’s Manual. Scientia Publishing, Budapest.

[B35] Podani J Schmera D (2011) A new conceptual and methodological framework for exploring and explaining pattern in presence-absence data. Oikos 120(11): 1625–1638. 10.1111/j.1600-0706.2011.19451.x

[B36] Proctor J Lee YF Langley AM Munro WRC Nelson T (1988) Ecological studies on Gunung Silam, a small ultrabasic mountain in Sabah, Malaysia. I. Environment, forest structure and floristics. Journal of Ecology 76: 320–340. 10.2307/2260596

[B37] Richards P (1932) Ecology. In: Verdoon FR (Ed.) Manual of Bryology. Martinus Nijhoff, The Hague.

[B38] Ruinen J (1961) The phyllosphere. I. An ecologically neglected milieu. Plant and Soil 15(2): 81–109. 10.1007/BF01347221

[B39] Sabah Forestry Department (2017) Fact Sheets of Forest Reserves in Sabah. Sabah Forestry Department 48 pp.

[B40] Smith EP (1982) Niche breadth, resource availability, and inference. Ecology 63(6): 1675–1681. 10.2307/1940109

[B41] Sonnleitner M Dullinger S Wanek W Zechmeister H (2009) Micro climatic patterns correlate with the distribution of epiphyllous bryophytes in a tropical lowland rain forest in Costa Rica. Journal of Tropical Ecology 25(3): 321–330. 10.1017/S0266467409006002

[B42] Suleiman M Akiyama H Mohamed H (2006) A revised catalogue of mosses reported from Borneo. The Journal of the Hattori Botanical Laboratory 99: 107–184.

[B43] Suleiman M Masundang DP Akiyama H (2017) The mosses of Crocker Park, Malaysian Borneo. PhytoKeys 88: 71–107. 10.3897/phytokeys.88.14674 PMC5672137 29118647

[B44] Tuomisto H Ruokolainen K Yli-Halla M (2003) Dispersal, environment, and floristic variation of western Amazonian forests. Science 299(5604): 241–244. 10.1126/science.1078037 12522248

[B45] Usui S Sato H Lee-Agama A Chua R (2006) Crocker Range Park Management Plan. Kota Kinabalu. Sabah Parks, 193 pp.

[B46] Wanek W Portl K (2005) Phyllosphere nitrogen relations: Reciprocal transfer of nitrogen between epiphyllous liverworts and host plants in tropical wet forests in Costa Rica. The New Phytologist 166: 577–588. 10.1111/j.1469-8137.2005.01319.x 15819919

[B47] Whittaker RH (1972) Evolution and measurement of species diversity. Taxon 21(2–3): 213–251. 10.2307/1218190

[B48] Winkler S (1967) Die epiphyllen Moose der Nebelwälder von El Salvador C. A. Revue Bryologique et Lichénologique 35: 303–369.

[B49] Winkler S (1970) Ökologische Beziehungen zwischen den epiphyllen Moosen der Regenwälder des Choco (Colombia, S.A.). Revue Bryologique et Lichénologique 37: 949–959.

[B50] Yamada K (1989) The bryophytes of Sabah (North Borneo) with special reference to the BRYOTROP transect of Mount Kinabalu. VIII. *Radula* (Radulaceae, Hepaticopsida). Willdenowia 19: 219–236.

[B51] Zhu RL Lei S Andi MAM Suleiman M (2017) *Thiersianthus* (Marchantiophyta: Lejeuneaceae), a new genus from lowland rainforests in Borneo. The Bryologist 120(4): 511–520. 10.1639/0007-2745-120.4.511

[B52] Zotz G Bude B Meyer A Zellner H Lange OL (1997) Water relations and CO_2_ exchange of tropical bryophytes in a lower montane rain forest in Panama. Botanica Acta 110(1): 9–17. 10.1111/j.1438-8677.1997.tb00605.x

